# Molecular epidemiology and antimicrobial resistance of human *Streptococcus suis* isolates in Guangxi, China, 2015–2021

**DOI:** 10.3389/fpubh.2026.1873768

**Published:** 2026-08-28

**Authors:** Xueli Yi, Bin Luo, Yuanyuan Dai, Ying Wei, Shimao Zhong, Ying Deng, Chunfang Wang, Yuanji Teng

**Affiliations:** 1 Affiliated Hospital of Youjiang Medical University for Nationalities, Guangxi, China; 2 Key Laboratory of Research on Clinical Molecular Diagnosis for High Incidence Diseases in Western Guangxi of Guangxi Higher Education Institutions, Education Department of Guangxi Zhuang Autonomous Region, Baise, Guangxi, China

**Keywords:** antimicrobial resistance, molecular epidemiology, multilocus sequence typing, serotype, *Streptococcus suis*, whole-genome sequencing

## Abstract

**Background:**

*Streptococcus suis* (*S. suis*) is an important zoonotic pathogen and a common colonizer of the upper respiratory tract of pigs. Human infections have been reported in several regions of China, including Guangxi, but genomic and antimicrobial resistance data from this region remain limited. This study investigated the molecular epidemiology, antimicrobial susceptibility, and genomic characteristics of human *S. suis* isolates collected in Baise City, Guangxi, from 2015 to 2021.

**Methods:**

This retrospective study included 39 non-duplicate clinical isolates confirmed as *S. suis* by whole-genome analysis. Antimicrobial susceptibility testing was performed using a broth microdilution-based system and interpreted according to the Clinical and Laboratory Standards Institute guidelines. Serotypes were determined by agglutination using type-specific antisera. Whole-genome sequencing was used for species confirmation, multilocus sequence typing, detection of antimicrobial resistance and virulence-associated genes, and core-protein phylogenetic analysis.

**Results:**

The median patient age was 55 years, and 36/39 (92.3%) patients were male. Meningitis was documented in 34/39 (87.2%) patients, and hearing impairment occurred in 21/39 (53.8%). Pig- or pork-related exposure was recorded in 15/39 (38.5%) patients. Resistance was highest to tetracycline (38/39, 97.4%), followed by erythromycin and clindamycin (26/39, 66.7% each). Four isolates (10.3%) showed intermediate susceptibility to penicillin, but none were resistant. All isolates remained susceptible to ampicillin, ceftriaxone, levofloxacin, linezolid, vancomycin, and meropenem. Serotype 2 predominated (32/39, 82.1%), followed by serotype 14 (7/39, 17.9%), while ST1 (29/39, 74.4%) and ST7 (7/39, 17.9%) were the two major sequence types. Resistance genes were mainly associated with tetracyclines, macrolides, lincosamides, and aminoglycosides. All ST1 isolates carried *mrp* and lacked *tet*(40), while all ST7 isolates showed the reverse pattern.

**Conclusion:**

Serotype 2 and ST1 predominated among the human *S. suis* isolates collected at this center. Resistance to tetracycline, erythromycin, and clindamycin was common, while susceptibility to the *β*-lactams tested was largely preserved. Differences in virulence- and resistance-associated gene profiles were also observed between the major lineages, indicating distinct genetic characteristics among the locally circulating isolates.

## 1 Introduction

*Streptococcus suis (S. suis)* is a Gram-positive bacterium and a common commensal of the upper respiratory tract, especially the nasopharynx and tonsils, of healthy pigs. Carriage is usually asymptomatic, but stress, co-infections, poor husbandry, or highly virulent serotypes can lead to invasive disease (1, 2). These infections contribute to mortality, treatment costs, and reduced productivity, making *S. suis* an important pathogen in the swine industry. The bacterium is widely distributed in pig populations across China. A systematic review and meta-analysis of studies published between 2000 and 2021 estimated an overall prevalence of 40.8% among pigs in China, with a pooled prevalence of approximately 50.6% in Guangxi (3).

*S. suis* is also an important zoonotic pathogen. Human infection usually causes severe invasive disease, most commonly meningitis, but may also present as sepsis, endocarditis, or septic arthritis (4). Human infections caused by *S. suis* are usually sporadic, but large outbreaks have occasionally occurred, especially those involving highly virulent strains (5).

Human infections with *S. suis* have been reported worldwide (6). In Southeast Asia, particularly in Thailand and Vietnam, *S. suis* is recognized as one of the leading causes of adult bacterial meningitis (7, 8). In China, two major outbreaks of human *S. suis* infection occurred in 1998 and 2005, resulting in significant morbidity and mortality (2, 5, 9). Sporadic human cases have since been reported in multiple provinces, with relatively frequent reports from Guangxi (10). In June 2016, a *S. suis* outbreak occurred in Guangxi, China, which was associated with occupational exposure to infected pigs and the handling or consumption of contaminated pork products. Six clinical isolates were collected during this outbreak, which was associated with one reported fatal case (11). Previous studies reported genetic relatedness between some human and porcine *S. suis* isolates collected during the same period, supporting the zoonotic potential of the pathogen (10). These findings underscore the ongoing public health relevance of *S. suis* in the region.

Antimicrobial resistance in *S. suis* has been increasingly reported worldwide (4). High resistance rates to tetracyclines, macrolides, lincosamides, and aminoglycosides have been observed in both human and porcine isolates, suggesting that *S. suis* may serve as an important reservoir of antimicrobial resistance genes and contribute to horizontal gene transfer across bacterial species (12, 13).

Despite previous reports of human infection in Guangxi, molecular epidemiological data from Baise City remain scarce. The present study sought to characterize the antimicrobial resistance patterns and molecular epidemiological features of human clinical isolates initially identified as *S. suis* in Baise City, Guangxi, from 2015 to 2021, with the aim of providing local evidence for surveillance in western Guangxi.

## 2 Materials and methods

### 2.1 Study design and study population

This retrospective study was conducted at the Affiliated Hospital of Youjiang Medical University for Nationalities, a tertiary teaching hospital in Baise City, western Guangxi, China. Samples were identified by reviewing clinical microbiology laboratory records for patients from whom isolates initially identified as *S. suis* were recovered between January 2015 and December 2021. A laboratory-confirmed invasive case was defined as a patient with a genome-confirmed *S. suis* isolate recovered from a normally sterile site (blood or cerebrospinal fluid) and clinical evidence of infection documented in the medical record. Only the first isolate recovered from each patient during the study period was included. For patients with positive cultures from both blood and cerebrospinal fluid (CSF), isolate source referred to the specimen yielding the first included isolate, whereas the culture-positive specimen category included all positive specimen types documented for that patient.

A total of 41 non-duplicate isolates were recovered from 41 patients and initially identified as *S. suis* using routine laboratory procedures. All isolates underwent whole-genome sequencing for species-level confirmation. Thirty nine isolates were confirmed as *S. suis*, while isolates 26 and D7 could not be conclusively assigned to this species and were excluded from the final study population. All subsequent epidemiological analyses, antimicrobial susceptibility testing, serotyping, multilocus sequence typing, virulence-associated gene analysis, antimicrobial resistance gene analysis, and phylogenetic analysis were therefore based on the 39 genome-confirmed *S. suis* isolates.

### 2.2 Bacterial isolation

Blood and CSF specimens were collected by trained clinical staff using aseptic techniques. Both specimen types were inoculated into blood culture bottles and transported to the clinical microbiology laboratory for processing. Before culture, patient identification, specimen type, collection details, and container integrity were checked according to the laboratory’s standard operating procedures (SOPs). For blood culture samples, the same bacterial species was recovered from both submitted bottles. The clinical significance of each culture result was assessed through review of the available microbiological and clinical records, and possible contamination was excluded. Because CSF is normally sterile, isolates recovered from CSF were considered clinically significant. Positive culture bottles were subcultured onto 5% sheep blood agar plates and incubated at 35 °C in 5% CO₂ for 24–48 h. Colonies suspected to belong to the genus *Streptococcus* were selected for further identification. All isolates were stored at −80 °C until subsequent analysis. All isolates were cultured and preserved in accordance with the *National Clinical Laboratory Operating Procedures* (4th edition, China).

### 2.3 Bacterial identification

All isolates were subcultured on 5% sheep blood agar plates and incubated at 35 °C in 5% CO₂ for 24 h. Preliminary identification was performed using the VITEK MS system (bioMérieux, Marcy-l’Étoile, France) in IVD mode with the VITEK MS Knowledge Base version 3.2. Instrument calibration and quality control were carried out according to the manufacturer’s instructions using *Escherichia coli* ATCC 8739 and *Klebsiella aerogenes* ATCC 13048. Identification results were accepted only when the quality control results met the manufacturer’s criteria.

For each isolate, a single colony was spotted onto a disposable target slide and overlaid with 1 μL of matrix solution (*α*-cyano-4-hydroxycinnamic acid), followed by air drying at room temperature prior to analysis. The VITEK MS system reports identification results as confidence values ranging from 0 to 99.9%. Only identifications classified by the system as acceptable at the species level were retained, whereas isolates with low discrimination or inconclusive results were subjected to repeat testing or further genomic confirmation (14, 15).

Whole-genome average nucleotide identity was used to confirm species identity. ANIb values were calculated in JSpeciesWS by comparing each assembled genome with the reference strains *S. suis* P1/7 and *S. parasuis* SUT-286. Only isolates showing species-level similarity to *S. suis* were retained.

### 2.4 Antimicrobial susceptibility testing

Antimicrobial susceptibility testing was performed using a commercial broth microdilution-based system for *Streptococcus* spp. (Zhuhai DL Biotech Co., Ltd., Zhuhai, China) according to the manufacturer’s instructions. Bacterial suspensions were prepared in sterile saline to a turbidity equivalent to a 2.0 McFarland standard and diluted in the manufacturer-provided inoculation medium. The standardized inoculum was dispensed into wells containing predefined concentrations of the antimicrobial agents, and the panels were incubated at 35 °C for 24 h. Minimum inhibitory concentrations were determined based on visible bacterial growth.

*Streptococcus pneumoniae* ATCC 49619 was used as the quality-control strain. The observed MIC values were compared with the acceptable quality-control ranges specified in CLSI M100, 35th edition, and all results were within the corresponding ranges (Supplementary Table S1). Antimicrobial susceptibility was interpreted using the MIC breakpoints for *Streptococcus* spp. viridans group in CLSI M100, 35th edition. Isolate-level MIC values and susceptibility interpretations for the 39 genome-confirmed *S. suis* isolates are presented in Supplementary Table S5, and the interpretive MIC breakpoints used for each antimicrobial agent are listed in Supplementary Table S6.

### 2.5 Molecular epidemiological analysis

#### 2.5.1 DNA extraction, whole-genome sequencing, and genome analysis

Genomic DNA was extracted from bacterial cultures using a commercial bacterial DNA extraction kit (Shanghai Tianhao Biotechnology Co., Ltd., Shanghai, China) according to the manufacturer’s instructions. Sequencing libraries were prepared using a standard Illumina library preparation protocol, and whole-genome sequencing (WGS) was performed on the Illumina NovaSeq platform (Illumina, San Diego, CA, USA), generating paired-end reads.

Raw sequencing data were subjected to standard quality control procedures to remove low-quality reads and adapter sequences. High-quality reads were *de novo* assembled using SPAdes (version 3.14) with default parameters. The target sequencing depth was approximately 100 × .

Genome assembly quality was assessed using QUAST (version 5.0.2) to evaluate assembly statistics, including N50 and number of contigs. Genome completeness and contamination were assessed using CheckM (version 1.1.3). Genome annotation was performed using Prokka (version 1.13.3) with default parameters. These metrics were used to ensure the reliability of genome assemblies for downstream analyses.

#### 2.5.2 Average nucleotide identity (ANI)

Average nucleotide identity was calculated using the BLAST-based ANIb method implemented in JSpeciesWS (https://jspecies.ribohost.com/jspeciesws/). The assembled genomes were compared with the reference genomes of *S. suis* P1/7 and *S. parasuis* SUT-286 using the default parameters of the platform (16, 17). Only isolates with ANI values supporting species-level assignment to *S. suis* were included in the final dataset. Isolates 26 and D7 could not be conclusively assigned at the species level and were excluded from all subsequent analyses.

#### 2.5.3 Multilocus sequence typing (MLST)

Multilocus sequence typing was performed using the *S. suis* scheme available on the PubMLST website. The sequences of the seven housekeeping genes, *aroA*, *cpn60*, *dpr*, *gki*, *mutS*, *recA*, and *thrA*, were submitted to the online database, and allele numbers and sequence types were assigned according to the corresponding allelic combinations (https://pubmlst.org/organisms/streptococcus-suis).

#### 2.5.4 Serotype determination

The 39 genome-confirmed *S. suis* isolates were serotyped by agglutination using type-specific antisera purchased from Statens Serum Institut (Copenhagen, Denmark), according to the manufacturer’s instructions. A serotype was assigned when a clear reaction was observed with the corresponding antiserum. Weak or inconclusive reactions were retested using fresh cultures, and isolates that remained untypeable after repeat testing were classified as nontypeable.

#### 2.5.5 Detection of virulence genes

Virulence-associated genes were identified by comparing assembled genome sequences against the Virulence Factor Database (VFDB, http://www.mgc.ac.cn/VFs/main.htm) using BLAST. The assembled genome sequences were first screened against VFDB to identify known virulence-associated genes. In addition, *S. suis*-specific virulence-associated genes reported in previous studies were compiled to construct a custom reference dataset comprising 91 genes (10). A gene was considered present when the alignment showed ≥90% sequence identity and ≥80% coverage.

The identified virulence-associated genes were grouped according to their putative functions into transcription factors/two-component systems (TCSs), enzymes/proteases, surface/secreted components, regulation, nutritional/metabolic factors, post-translational modification, adherence, stress survival, immune modulation, and other functions, based on previous studies of *S. suis* and available database annotations. Because some genes may have multiple biological functions, these categories were used primarily to facilitate descriptive interpretation of the gene profiles.

#### 2.5.6 Detection of antimicrobial resistance genes

Assembled sequences were uploaded to the ResFinder database (https://cge.food.dtu.dk/services/ResFinder/) to identify antimicrobial resistance genes. Genes with >95% coverage and >99% identity were considered present.

#### 2.5.7 Core-protein phylogenetic analysis

Core-protein phylogenetic analysis was performed for the 39 genome-confirmed *S. suis* isolates. Genome annotation was carried out using Prokka (version 1.13.3), and orthologous genes were clustered using Roary (version 3.12.0) with a minimum sequence identity threshold of 85%. This threshold was used only for ortholog clustering. Genes present in all 39 genomes (100% prevalence) were defined as core genes. Using this criterion, 1,583 core genes were identified and included in the phylogenetic analysis. The corresponding core protein sequences were aligned using MAFFT (version 7.407) and concatenated into a single alignment. Poorly aligned and unreliable regions were removed using Gblocks (version 0.91b). The best-fitting amino acid substitution model was selected in IQ-TREE 2 according to the Bayesian information criterion. A maximum-likelihood tree was then constructed using the selected model, and branch support was assessed with 1,000 bootstrap replicates. The tree was visualized and annotated using the Interactive Tree of Life (iTOL). Isolates 26 and D7 were excluded from the phylogenetic analysis because their species assignments remained unresolved and they were not included in the final *S. suis* dataset.

### 2.6 Statistical analysis

Statistical analyses were performed using IBM SPSS Statistics version 29.0 (IBM Corp., Armonk, NY, USA). WHONET version 5.6 was used for the management and analysis of antimicrobial susceptibility data. Continuous variables were summarized as medians with interquartile ranges (IQRs), and categorical variables as counts and percentages. Gene carriage rates were compared between serotype 2 and serotype 14 isolates and between ST1 and ST7 isolates using Fisher’s exact test. For gene-level comparisons, *p* values were adjusted for multiple testing using the Benjamini–Hochberg false discovery rate (FDR) procedure. FDR correction was performed separately for the 70 virulence-associated genes and the 19 antimicrobial resistance genes. A two-sided *p* value < 0.05 was considered statistically significant, and statistical significance after multiple-comparison correction was defined as an FDR-adjusted *p* value < 0.05.

## 3 Results

### 3.1 Epidemiological and clinical characteristics of the patients

The final cohort comprised 39 patients with genome-confirmed *S. suis* infection. The median age was 55 years (IQR, 46.5–63.5; range, 23–89), and 36 patients (92.3%) were male. Four patients (10.3%) were younger than 40 years, 20 (51.3%) were aged 40–59 years, and 15 (38.5%) were aged 60 years or older.

The study isolates were recovered from cerebrospinal fluid in 25 patients (64.1%) and from blood in 14 (35.9%). Review of all available culture records showed that 23 patients (59.0%) had positive cerebrospinal fluid cultures only, 13 (33.3%) had positive blood cultures only, and three (7.7%) had both specimens positive. Meningitis was documented in 34 patients (87.2%), with one additional probable case.

Pig- or pork-related exposure was recorded in 15 patients (38.5%). Thirty-six patients (92.3%) improved and were discharged, while three (7.7%) were transferred; no in-hospital deaths were recorded. Hearing impairment was documented in 21 patients (53.8%), low back pain in nine (23.1%), and joint pain in seven (17.9%). Among 38 patients included in the length-of-stay analysis, the median hospital stay was 19.0 days (IQR, 14.3–24.0; range, 7–52). These findings are summarized in Table 1.

**Table 1:** Demographic and clinical characteristics of the 39 patients with genome-confirmed *Streptococcus suis* infection.

Clinical characteristic	No. of patients	Value
Age, years		
Median (IQR)		55 (46.5–63.5)
Range		23–89
Age group, years		
<40	4	4/39 (10.3%)
40–59	20	20/39 (51.3%)
≥60	15	15/39 (38.5%)
Sex		
Male	36	36/39 (92.3%)
Female	3	3/39 (7.7%)
Isolate source		
Cerebrospinal fluid	25	25/39 (64.1%)
Blood	14	14/39 (35.9%)
Culture-positive specimen(s)		
Cerebrospinal fluid only	23	23/39 (59.0%)
Blood only	13	13/39 (33.3%)
Both cerebrospinal fluid and blood	3	3/39 (7.7%)
Clinical diagnosis		
Meningitis	34	34/39 (87.2%)
Probable meningitis	1	1/39 (2.6%)
No documented diagnosis of meningitis	3	3/39 (7.7%)
Diagnosis uncertain	1	1/39 (2.6%)
Documented pig or pork exposure	15	15/39 (38.5%)
Clinical outcome		
Improved and discharged	36	36/39 (92.3%)
Transferred to another hospital	3	3/39 (7.7%)
In-hospital death	0	0/39 (0%)
Major complications or sequelae		
Hearing impairment	21	21/39 (53.8%)
Low back pain	9	9/39 (23.1%)
Joint pain	7	7/39 (17.9%)
Hospital stay, days		
Median (IQR)	38	19.0 (14.3–24.0)
Range	38	7–52

IQR, interquartile range.

### 3.2 Species identification

All 41 isolates were initially identified as *S. suis* by VITEK MS. ANIb analysis confirmed 39 isolates as *S. suis*, with ANI values of 96.81–99.99% against the reference strain *S. suis* P1/7. Isolates 26 and D7 showed much lower ANI values against *S. suis* P1/7 (82.68 and 82.69%, respectively) but higher values against *S. parasuis* SUT-286 (94.47 and 94.46%). However, their ANI values remained below the commonly accepted 95–96% threshold for species-level assignment (18, 19). The identities of the two isolates were therefore considered unresolved, and both were excluded from the final study population and all downstream analyses. Species-identification results for the 41 initially screened isolates are summarized in Table 2, while isolate-level ANI values and genome accession numbers are provided in Supplementary Table S2. The final analytical dataset consisted of 39 genome-confirmed *S. suis* isolates.

**Table 2:** Species-identification results for the 41 initially screened clinical isolates.

Isolate group or ID	No. of isolates	Initial identification by VITEK MS	ANI (%) vs. *S. suis* P1/7	ANI (%) vs. *S. parasuis* SUT-286	Final species assignment
Genome-confirmed *S. suis* isolates	39	*S. suis*	96.81–99.99	83.63–84.11	*S. suis*
Isolate 26	1	*S. suis*	82.68	94.47	Unresolved
Isolate D7	1	*S. suis*	82.69	94.46	Unresolved

Isolates 26 and D7 showed greater genomic similarity to *S. parasuis* SUT-286 than to *S. suis* P1/7. However, their ANI values did not reach the commonly accepted species-level threshold (95–96%). Their species assignment was considered unresolved, and they were excluded from the final study population and all downstream analyses.

### 3.3 Antimicrobial susceptibility profiles

All 39 *S. suis* isolates were susceptible to ampicillin, ceftriaxone, levofloxacin, linezolid, vancomycin, and meropenem. Thirty-five isolates (89.7%) were susceptible to penicillin, while four (10.3%) showed intermediate susceptibility; none were resistant. Resistance was highest to tetracycline, affecting 38 isolates (97.4%), followed by erythromycin and clindamycin, with 26 isolates (66.7%) resistant to each agent. Overall susceptibility results are summarized in Table 3. Isolate-level MIC values and interpretations are provided in Supplementary Table S5, and the MIC breakpoints used for interpretation are listed in Supplementary Table S6.

**Table 3:** Antimicrobial susceptibility profiles of the 39 clinical isolates.

Antimicrobial agent	Susceptible, *n* (%)	Intermediate, *n* (%)	Resistant, *n* (%)
Ampicillin	39 (100.0)	0 (0)	0 (0)
Ceftriaxone	39 (100.0)	0 (0)	0 (0)
Clindamycin	13 (33.3)	0 (0)	26 (66.7)
Erythromycin	13 (33.3)	0 (0)	26 (66.7)
Levofloxacin	39 (100.0)	0 (0)	0 (0)
Linezolid	39 (100.0)	0 (0)	0 (0)
Tetracycline	1 (2.6)	0 (0)	38 (97.4)
Penicillin	35 (89.7)	4 (10.3)	0 (0)
Vancomycin	39 (100.0)	0 (0)	0 (0)
Meropenem	39 (100.0)	0 (0)	0 (0)

*n*, number of isolates. Antimicrobial susceptibility results were interpreted according to CLSI M100, 35th edition.

### 3.4 Serotype distribution

**Figure 1:**
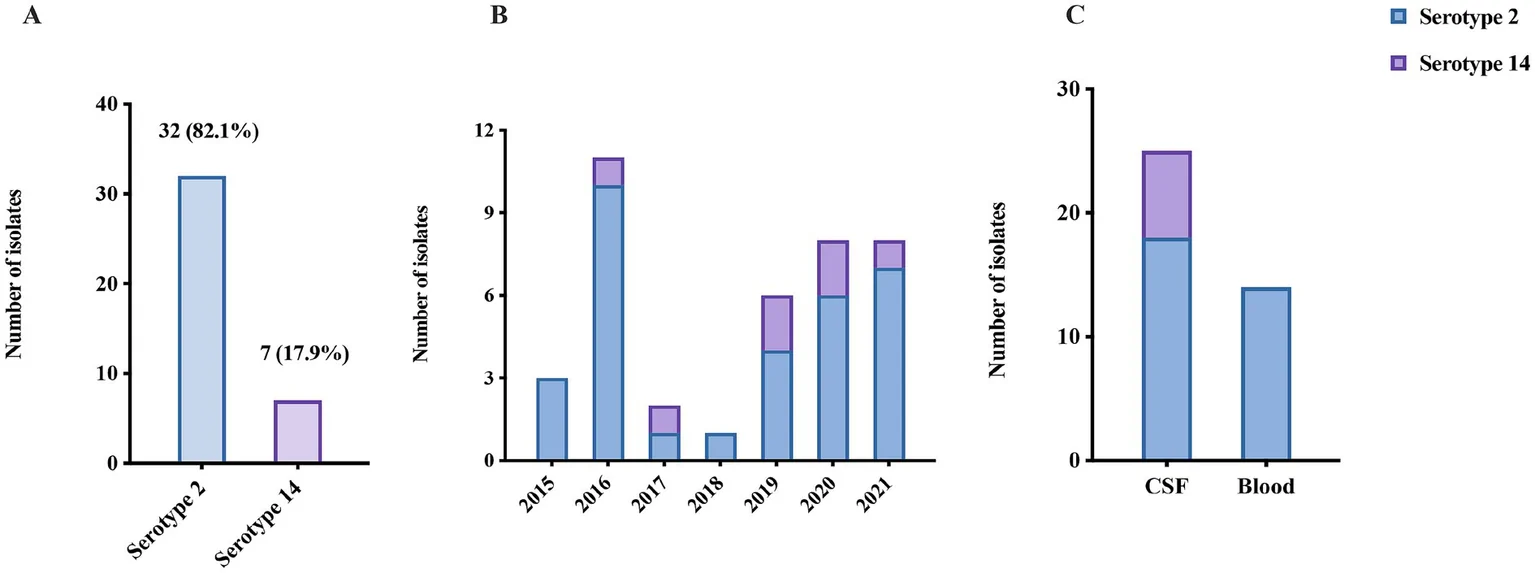
Distribution of serotypes among the 39 genome-confirmed *S. suis* isolates. **(A)** Overall distribution of serotypes 2 and 14. Numbers above the bars indicate the number and percentage of isolates. **(B)** Distribution of serotypes by year of isolation. **(C)** Distribution of serotypes by specimen source. Isolates 26 and D7 were excluded from the serotype analysis because they were not included in the final *S. suis* dataset. CSF, cerebrospinal fluid.

Among the 39 genome-confirmed *S. suis* isolates, 32 (82.1%) were identified as serotype 2 and seven (17.9%) as serotype 14. Serotype 2 was recovered in every year of the study period, whereas serotype 14 was detected in 2016, 2017, 2019, 2020, and 2021. All seven serotype 14 isolates were recovered from cerebrospinal fluid. The distribution of serotypes overall, by year, and by specimen source is shown in Figure 1, with isolate-level details provided in Supplementary Table S3.

### 3.5 Multilocus sequence typing

**Figure 2:**
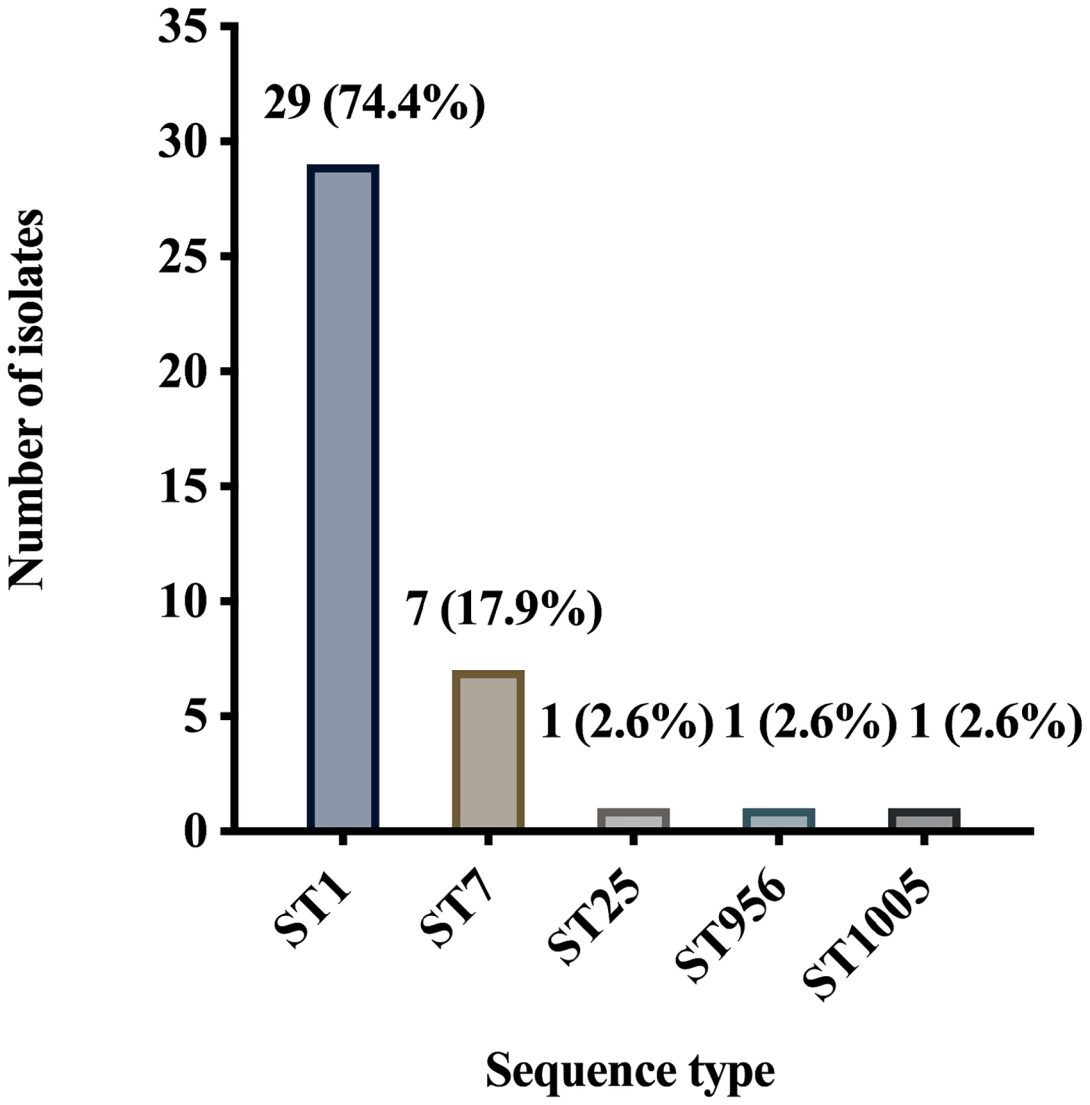
Distribution of sequence types among the 39 genome-confirmed *S. suis* isolates. Bars indicate the number of isolates assigned to each sequence type. Labels above the bars show the number and percentage of isolates. Isolates 26 and D7 were excluded from the *S. suis* MLST analysis because their species assignments remained unresolved.

MLST identified five sequence types among the 39 genome-confirmed *S. suis* isolates. ST1 was the most common, comprising 29 isolates (74.4%), followed by ST7 with seven isolates (17.9%). ST25, ST956, and ST1005 were each represented by a single isolate (2.6%). The distribution of sequence types is shown in Figure 2, and isolate-level results are provided in Supplementary Table S3.

### 3.6 Virulence gene profiles

**Figure 3:**
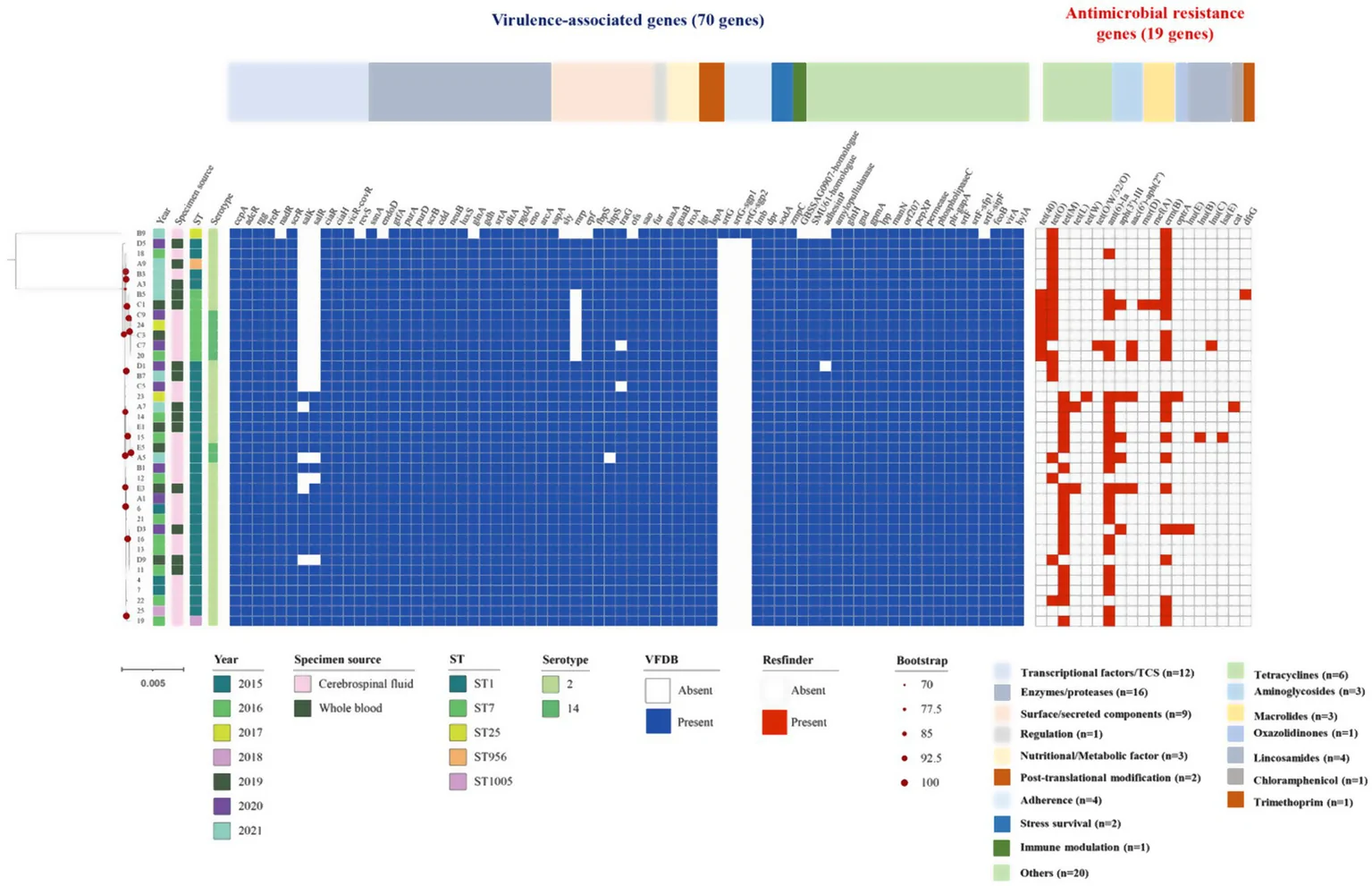
Core-protein phylogeny and distribution of virulence-associated and antimicrobial resistance genes among 39 *Streptococcus suis* isolates. The core-protein maximum-likelihood phylogenetic tree is shown together with isolate metadata, including year of isolation, specimen source, sequence type (ST), and serotype. The adjacent heatmaps show the presence or absence of 70 virulence-associated genes identified through VFDB screening and a literature-curated *S. suis*-specific reference dataset, together with 19 antimicrobial resistance genes identified using ResFinder. Virulence-associated genes are grouped according to their functional categories, and antimicrobial resistance genes are grouped by antimicrobial class, as indicated by the annotation bars above the heatmaps. Blue and white cells indicate the presence and absence of virulence-associated genes, respectively; red and white cells indicate the presence and absence of antimicrobial resistance genes, respectively. Colored circles at the tree nodes indicate bootstrap support, with circle size proportional to the bootstrap value. The scale bar represents the number of substitutions per site.

Virulence-associated genes were widely distributed among the 39 *S. suis* isolates, although their carriage patterns varied across isolates and lineages (Figure 3). The detected genes spanned several functional categories, including transcription factors and two-component systems, enzymes and proteases, surface or secreted components, regulation, nutritional and metabolic factors, post-translational modification, adherence, stress survival, immune modulation, and other functions. Several genes, including *eno, gdh, dltA, sodA, adcR,* and *gpmA*, were present in most isolates, whereas the pilus-associated genes *srtG, srtG-sgp1,* and *srtG-sgp2* were detected only in isolate B9.

**Figure 4:**
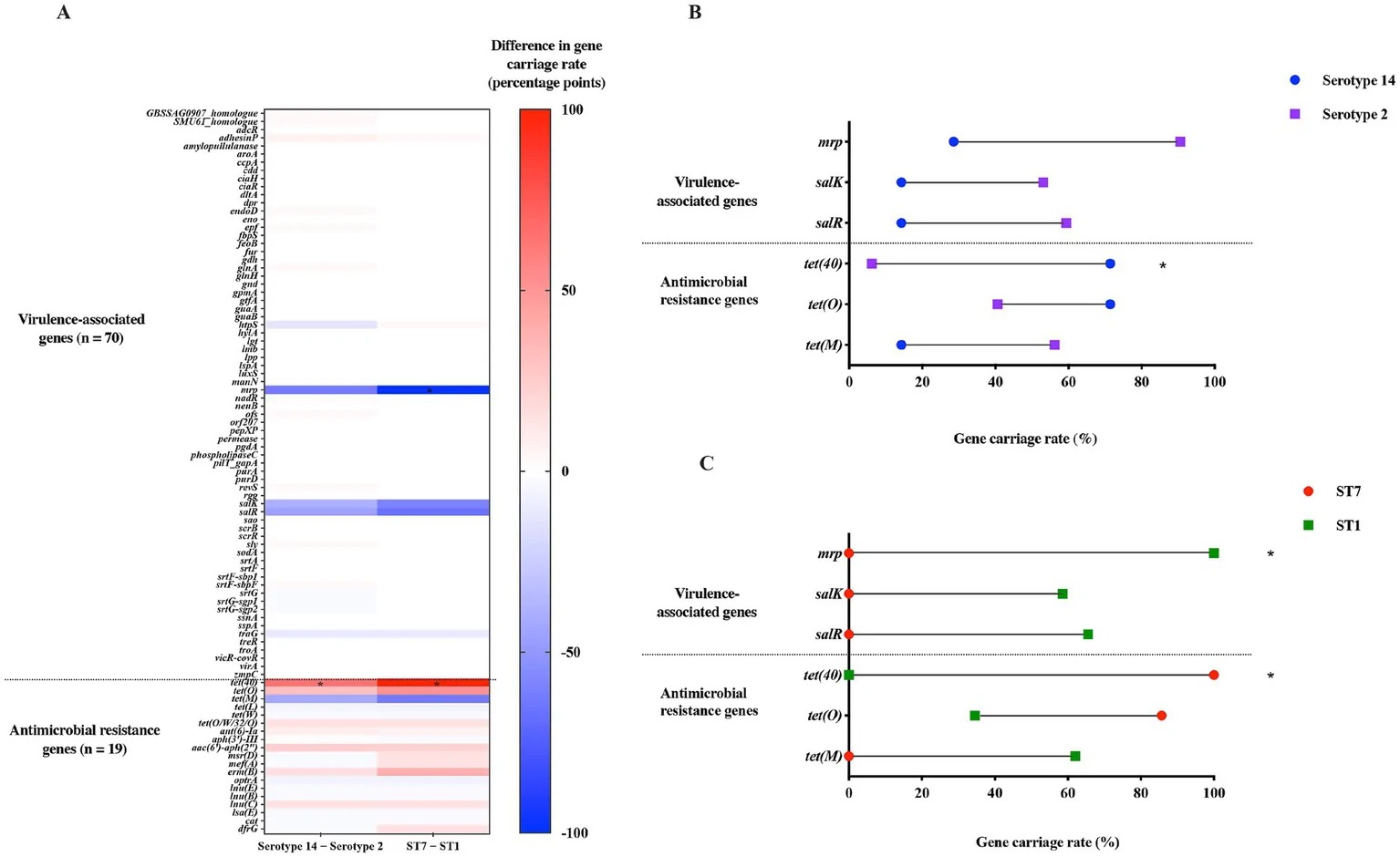
Comparison of virulence-associated and antimicrobial resistance gene carriage among major serotypes and sequence types of *Streptococcus suis*. **(A)** Heatmap showing differences in the carriage rates of 70 virulence-associated genes and 19 antimicrobial resistance genes between serotype 14 and serotype 2 isolates and between ST7 and ST1 isolates. Differences are expressed as percentage points. Positive values indicate higher carriage in serotype 14 or ST7, whereas negative values indicate higher carriage in serotype 2 or ST1. **(B)** Dumbbell plot showing the carriage rates of six selected genes with notable differences between serotype 14 and serotype 2 isolates. **(C)** Dumbbell plot showing the carriage rates of the same six genes between ST7 and ST1 isolates. The selected genes comprise three virulence-associated genes (*mrp, salK,* and *salR*) and three antimicrobial resistance genes [*tet(40), tet(O),* and *tet(M)*]. In panels B and C, each line connects the carriage rates of the two comparison groups for the same gene. Asterisks indicate statistically significant differences after FDR correction (FDR-adjusted *p* < 0.05).

Comparison of the two major serotypes and sequence types showed that several virulence-associated genes differed in carriage frequency (Figure 4; Table 4). *mrp* was detected in 29/32 (90.6%) serotype 2 isolates and 2/7 (28.6%) serotype 14 isolates (*p* = 0.002), although this difference did not remain significant after FDR correction (FDR-adjusted *p* = 0.122). A stronger lineage-associated pattern was observed between ST1 and ST7: *mrp* was present in all ST1 isolates (29/29, 100.0%) but absent from all ST7 isolates (0/7, 0.0%; *p* < 0.001; FDR-adjusted *p* < 0.001). *salR* and *salK* were also more frequently detected in ST1 than in ST7, but these differences were no longer significant after FDR correction. Complete gene-level comparisons are provided in Supplementary Table S7.

**Table 4:** Comparison of selected virulence-associated and antimicrobial resistance gene carriage between the major serotypes and sequence types of *Streptococcus suis* isolates.

Gene	Gene category	Functional/Antimicrobial class	Serotype comparison				Sequence-type comparison			
			Serotype 2, *n*/*N* (%)	Serotype 14, *n*/*N* (%)	*P*	FDR-adjusted *P*	ST1, *n*/*N* (%)	ST7, *n*/*N* (%)	*P*	FDR-adjusted *P*
*tet(40)*	Antimicrobial resistance	Tetracyclines	2/32 (6.2%)	5/7 (71.4%)	0.001	0.013	0/29 (0.0%)	7/7 (100.0%)	<0.001	<0.001
*tet(M)*	Antimicrobial resistance	Tetracyclines	18/32 (56.2%)	1/7 (14.3%)	0.091	0.682	18/29 (62.1%)	0/7 (0.0%)	0.008	0.072
*tet(O)*	Antimicrobial resistance	Tetracyclines	13/32 (40.6%)	5/7 (71.4%)	0.215	0.682	10/29 (34.5%)	6/7 (85.7%)	0.030	0.189
*mrp*	Virulence-associated	Surface/secreted components (10)	29/32 (90.6%)	2/7 (28.6%)	0.002	0.122	29/29 (100.0%)	0/7 (0.0%)	<0.001	<0.001
*salR*	Virulence-associated	Transcriptional factors/TCS (10)	19/32 (59.4%)	1/7 (14.3%)	0.044	1.000	19/29 (65.5%)	0/7 (0.0%)	0.002	0.082
*salK*	Virulence-associated	Transcriptional factors/TCS (10)	17/32 (53.1%)	1/7 (14.3%)	0.098	1.000	17/29 (58.6%)	0/7 (0.0%)	0.008	0.195

Data are presented as *n*/*N* (%), *n* represents the number of isolates carrying the indicated gene and *N* represents the total number of isolates in the corresponding serotype or sequence-type group. For gene-level comparisons, P values were adjusted using the Benjamini–Hochberg false discovery rate (FDR) procedure, with FDR correction performed separately for the 70 virulence-associated genes and the 19 antimicrobial resistance genes. An FDR-adjusted *p*=value <0.05 was considered statistically significant.

ST, sequence type; FDR, false discovery rate.

### 3.7 Antimicrobial resistance genes

Antimicrobial resistance genes were mainly associated with tetracyclines, aminoglycosides, macrolides, and lincosamides (Figure 3). Frequently detected genes included *ant(6)-Ia, erm(B), tet(M),* and *tet(O)*. Aminoglycoside resistance-associated genes, including *ant(6)-Ia, aph(3′)-III,* and *aac(6′)-aph(2″)*, were identified in multiple isolates; however, aminoglycoside susceptibility was not tested, and their presence was therefore not interpreted as evidence of phenotypic resistance.

Among the resistance genes compared between the major groups, *tet(40)* showed the most pronounced difference (Figure 4; Table 4). It was detected in 5/7 (71.4%) serotype 14 isolates but only 2/32 (6.2%) serotype 2 isolates (*p* = 0.001; FDR-adjusted *p* = 0.013). The lineage-associated difference was even stronger: all seven ST7 isolates carried *tet(40)*, whereas none of the 29 ST1 isolates did (*p* < 0.001; FDR-adjusted *p* < 0.001). *tet(M)* and *tet(O)* also differed between ST1 and ST7 in the unadjusted analyses, but neither remained significant after FDR correction. Complete comparisons for all resistance genes are provided in Supplementary Table S7.

Resistance genotypes were shown in Figure 3. Genotype–phenotype concordance was 92.3% for clindamycin, 92.3% for erythromycin, and 97.4% for tetracycline. Isolates 13, 24, and B7 were resistant to both clindamycin and erythromycin despite the absence of corresponding acquired resistance genes, whereas isolate 25 was resistant to tetracycline without a detectable tetracycline resistance gene. Isolate C5 carried no acquired resistance genes. Detailed isolate-level genotype–phenotype comparisons are provided in Supplementary Table S4.

### 3.8 Core-protein phylogenetic analysis

Core-protein phylogenetic analysis of the 39 *S. suis* isolates identified several lineages that broadly corresponded to sequence type (Figure 3). Most ST1 isolates clustered within the predominant lineage, whereas the ST7 isolates formed a separate group. The single ST25, ST956, and ST1005 isolates occupied distinct branches. Serotype 2 was predominantly associated with ST1, while serotype 14 was more frequently represented among ST7 isolates. Isolates from different years and specimen sources were distributed throughout the tree, with no clear temporal or specimen-specific clustering.

When considered alongside the phylogenetic analysis, the virulence and resistance gene profiles showed a clear lineage-associated pattern. All ST1 isolates carried *mrp* and lacked *tet(40)*, whereas all ST7 isolates showed the opposite pattern, lacking *mrp* but carrying *tet(40)*. The *salK* and *salR* genes were also absent from ST7 isolates but were present in a substantial proportion of ST1 isolates. These lineage-associated patterns were consistent with the statistical comparisons shown in Figure 4.

## 4 Discussion

*Streptococcus suis* is an important zoonotic pathogen capable of causing severe invasive infections in humans. The disease occurs worldwide but is particularly prevalent in Southeast Asia, where local climatic conditions, dietary habits, and occupational exposure may contribute to its transmission (20, 21). Clinically, *S. suis* infection can lead to a variety of severe conditions, including endocarditis, meningitis, sepsis, disseminated intravascular coagulation (DIC), suppurative arthritis, and endophthalmitis, with meningitis being the most common clinical manifestation (22). In China, several human outbreaks caused by highly pathogenic *S. suis* serotype 2 strains have been reported, most notably the outbreaks in Jiangsu Province in 1998 and Sichuan Province in 2005, highlighting the significant public health impact of this pathogen (5, 9).

In this study, we investigated the molecular epidemiology, antimicrobial resistance patterns, and genomic characteristics of human-derived *S. suis* isolates collected in Baise City, Guangxi, between 2015 and 2021. Serotype 2 and sequence type ST1 predominated among the isolates, consistent with previous reports identifying serotype 2 as the leading cause of human *S. suis* infections worldwide (4). Human *S. suis* infection may cause severe disease, including meningitis, sepsis, and streptococcal toxic shock-like syndrome (5, 10). However, serotype alone should not be regarded as evidence of increased virulence. We observed high resistance rates to tetracycline, erythromycin, and clindamycin, together with frequent carriage of tetracycline- and macrolide-associated resistance genes. These findings describe the antimicrobial resistance profile of the isolates collected at this center.

Guangxi is located in southern China, adjacent to Southeast Asian regions where human *S. suis* infections are frequently reported. The serotype and sequence-type distributions observed in this study provide local epidemiological information for western Guangxi. In southern Vietnam, serotype 2 predominated among human isolates from adults with meningitis, and an ST1-associated clonal group was also identified (22). However, the present study included only human isolates from Guangxi and did not include comparative genomes from Vietnam, other regions of China, or animal sources. Therefore, no conclusions can be drawn regarding cross-border transmission, regional circulation, or direct relationships between human and porcine lineages. Broader comparative genomic studies including human and animal isolates from multiple regions are needed to assess their evolutionary relationships.

Consistent with previous epidemiological studies, most patients in our cohort were middle-aged or older men (23). Cerebrospinal fluid was the most common specimen source, reflecting the fact that *S. suis* infection most frequently presents as bacterial meningitis in humans (24). The predominance of male patients has also been reported in several earlier studies and is generally attributed to occupational exposure (25). People engaged in pig farming, slaughtering, or processing raw pork have an elevated risk of infection through contact with infected animals or contaminated tissues (2). Consumption of raw or undercooked pork products is also a recognized risk factor for human *S. suis* infection (26, 27).

In our study, multilocus sequence typing revealed that ST1 was the predominant sequence type among the isolates, whereas ST7 represented the second most common lineage. ST7 strains have previously been associated with large-scale outbreaks of human *S. suis* infection in China and are generally considered to possess enhanced virulence (28, 29). Interestingly, the predominance of ST1 observed in Baise differs from the molecular epidemiological patterns reported in several other regions of China, where ST7 has often been reported as the dominant lineage. ST1 and ST7 differ at a single locus within the MLST scheme, suggesting that these sequence types are closely related but may represent distinct evolutionary lineages (30).

The *S. suis* infections that occurred in China in 1998 and 2005 were caused by serotype 2 ST7 strains, which have been associated with outbreaks in China and reported to harbor an approximately 89-kb pathogenicity island (89 K PAI) (31). In contrast, some sporadic ST7 strains have been described as lacking the 89 K PAI while carrying an inserted approximately 128-kb ICE–phage tandem mobile genetic element (32). Ji et al. reported that, among 33 human *S. suis* isolates collected in Shenzhen between 2005 and 2021, serotype 2 predominated, followed by serotype 14, while ST7 and ST1 accounted for 48.48 and 39.40% of the isolates, respectively (33). In the present collection, ST1 accounted for 29 of the 39 genome-confirmed *S. suis* isolates (74.4%). The sequence-type distribution in this single-center collection differed from that reported in the Shenzhen study.

Genome-based screening revealed differences in the distribution of predicted virulence-associated genes across the major lineages. Genes involved in adhesion, cell-envelope adaptation, resistance to oxidative stress, nutrient acquisition, and regulatory processes were widely distributed among the *S. suis* isolates. Notably, *mrp*, *salK*, and *salR* were absent from all ST7 isolates but were present in most ST1 isolates, indicating differences in gene content between these lineages. These findings should be interpreted cautiously. The presence of a virulence-associated gene does not establish its expression or contribution to pathogenicity, and several of the detected genes also have broader physiological or regulatory functions. Further functional studies are needed to determine whether these genomic differences are reflected in virulence-related phenotypes.

Antimicrobial susceptibility testing showed that most isolates remained susceptible to *β*-lactam antibiotics, which continue to serve as the primary therapeutic option for *S. suis* infections (20). Resistance was highest to tetracycline, followed by erythromycin and clindamycin. Resistance genes associated with tetracyclines and macrolides were frequently detected. These findings are consistent with previous reports describing widespread resistance to tetracyclines and macrolides among *S. suis* isolates from both human and animal sources (34, 35). The high prevalence of resistance to these antimicrobial classes may be related to their long-term and extensive use in veterinary practice (36, 37). Previous studies have shown that *S. suis* can carry transferable resistance elements, raising concern about its possible role as a reservoir of antimicrobial resistance genes (38).

Genomic analysis further showed that most isolates carried resistance-associated genes linked to more than one antimicrobial class. This finding describes the resistance-gene profiles of the isolates but does not establish phenotypic multidrug resistance. Although aminoglycoside susceptibility was not tested, aminoglycoside resistance-associated genes were detected in a substantial proportion of isolates, particularly *ant(6)-Ia*, which was frequently detected in this collection. Therefore, no conclusion regarding phenotypic aminoglycoside resistance could be drawn from gene carriage alone. The *tet*(40) gene was detected only in ST7 isolates in this collection. Given the small number of ST7 isolates, this pattern requires confirmation in larger datasets.

Several limitations should be noted. The number of isolates was relatively small, and all were collected from a single geographic region. Virulence- and resistance-associated determinants were identified through genome-based prediction, without experimental validation of their expression or biological roles. Targeted analysis of chromosomal variation in penicillin-binding protein genes was not performed; therefore, the genetic basis of the intermediate penicillin susceptibility observed in four isolates could not be determined.

Larger multicenter studies with more complete clinical data and experimental validation are therefore needed to better define the pathogenic mechanisms and epidemiological features of *S. suis*. Despite these limitations, the present study provides regional data on the molecular epidemiology and antimicrobial resistance of human-derived *S. suis* isolates in Guangxi and supports the need for continued surveillance to strengthen early warning and prevention efforts.

## 5 Conclusion

This retrospective, single-center study described the epidemiological, antimicrobial susceptibility, and genomic characteristics of 39 genome-confirmed human *Streptococcus suis* isolates collected in Baise City, Guangxi, between 2015 and 2021. Serotype 2 and ST1 predominated, while serotype 14 and several less common sequence types were also identified. Resistance was highest to tetracycline, erythromycin, and clindamycin, whereas susceptibility to the *β*-lactams tested was largely retained. Resistance-associated genes were mainly linked to tetracyclines, macrolides, lincosamides, and aminoglycosides, although aminoglycoside susceptibility was not assessed. Differences in virulence- and resistance-associated gene profiles were observed among the major lineages. These findings provide local data on the molecular epidemiology and antimicrobial resistance of human *S. suis* in western Guangxi and support continued surveillance.

## Data Availability

The datasets presented in this study can be found in online repositories. The names of the repository/repositories and accession number(s) can be found in the article/Supplementary material.
