# Supplementary material for: Molecular epidemiology and antimicrobial resistance of human Streptococcus suis isolates in Guangxi, China, 2015–2021
Source: Front Public Health. 2026 Aug 28;14:1873768. doi: 10.3389/fpubh.2026.1873768 (PMC13564376; doi:10.3389/fpubh.2026.1873768)
Supplement: Supplementary file 1 [file Table_1.docx]

Supplementary Table S1. Quality-control results for antimicrobial susceptibility testing

| Antimicrobial agent | CLSI acceptable MIC range (μg/mL) | Observed MIC (μg/mL) |
| --- | --- | --- |
| Ampicillin | 0.06–0.25 | 0.25 |
| Ceftriaxone | 0.03–0.12 | 0.12 |
| Clindamycin | 0.03–0.12 | 0.12 |
| Erythromycin | 0.03–0.12 | 0.12 |
| Levofloxacin | 0.5–2 | 0.5 |
| Linezolid | 0.25–2 | 0.25 |
| Tetracycline | 0.06–0.5 | 0.5 |
| Penicillin | 0.25–1 | 0.25 |
| Vancomycin | 0.12–0.5 | 0.12 |
| Meropenem | 0.03–0.25 | 0.25 |

**Abbreviations:** MIC, minimum inhibitory concentration; CLSI, Clinical and Laboratory Standards Institute. Streptococcus pneumoniae ATCC 49619 was used as the quality-control strain. Acceptable MIC ranges were based on CLSI M100, 35th edition.

Supplementary Table S2. Isolate-level average nucleotide identity and genome accession information

| Isolate ID | Sample ID | Genome  accession | ANI (%) *vs.*  S. suis P1/7 | ANI (%) *vs.*  *S. parasuis* SUT-286 | Final species assignment |
| --- | --- | --- | --- | --- | --- |
| 4 | SS01 | JBZGMP000000000 | 99.97 | 84.10 | *S. suis* |
| 6 | SS02 | JBZGMO000000000 | 99.97 | 84.10 | *S. suis* |
| 7 | SS03 | JBZGMN000000000 | 99.98 | 84.10 | *S. suis* |
| 11 | SS04 | JBZGMM000000000 | 99.92 | 84.11 | *S. suis* |
| 12 | SS05 | JBZGML000000000 | 99.97 | 83.99 | *S. suis* |
| 13 | SS06 | JBZGMK000000000 | 99.93 | 84.10 | *S. suis* |
| 14 | SS07 | JBZGMJ000000000 | 99.98 | 84.10 | *S. suis* |
| 15 | SS08 | JBZGMI000000000 | 99.92 | 84.08 | *S. suis* |
| 16 | SS09 | JBZGMH000000000 | 99.92 | 84.11 | *S. suis* |
| 18 | SS10 | JBZGMG000000000 | 99.97 | 83.99 | *S. suis* |
| 19 | SS11 | JBZGMF000000000 | 99.91 | 84.09 | *S. suis* |
| 20 | SS12 | JBZGME000000000 | 99.87 | 84.06 | *S. suis* |
| 21 | SS13 | JBZGMD000000000 | 99.96 | 84.10 | *S. suis* |
| 22 | SS14 | JBZGMC000000000 | 99.95 | 84.10 | *S. suis* |
| 23 | SS15 | JBZGMB000000000 | 99.97 | 84.08 | *S. suis* |
| 24 | SS16 | JBZGMA000000000 | 99.87 | 84.01 | *S. suis* |
| 25 | SS17 | JBZGLZ000000000 | 99.96 | 84.11 | *S. suis* |
| 26 | SS18 | JBZGLY000000000 | 82.68 | 94.47 | Unresolved |
| D7 | SS19 | JBZGLX000000000 | 82.69 | 94.46 | Unresolved |
| C3 | SS20 | JBZGLW000000000 | 99.80 | 84.00 | *S. suis* |
| E5 | SS21 | JBZGLV000000000 | 99.88 | 83.96 | *S. suis* |
| E3 | SS22 | JBZGLU000000000 | 99.96 | 84.05 | *S. suis* |
| D9 | SS23 | JBZGLT000000000 | 99.91 | 83.98 | *S. suis* |
| E1 | SS24 | JBZGLS000000000 | 99.91 | 84.08 | *S. suis* |
| C1 | SS25 | JBZGLR000000000 | 99.91 | 83.95 | *S. suis* |
| C5 | SS26 | JBZGLQ000000000 | 99.97 | 84.00 | *S. suis* |
| C7 | SS27 | JBZGLP000000000 | 99.87 | 83.98 | *S. suis* |
| C9 | SS28 | JBZGLO000000000 | 99.87 | 84.02 | *S. suis* |
| B1 | SS29 | JBZGLN000000000 | 99.97 | 84.07 | *S. suis* |
| D1 | SS30 | JBZGLM000000000 | 99.99 | 83.78 | *S. suis* |
| D3 | SS31 | JBZGLL000000000 | 99.91 | 83.93 | *S. suis* |
| D5 | SS32 | JBZGLK000000000 | 99.90 | 83.65 | *S. suis* |
| A1 | SS33 | JBZGLJ000000000 | 99.98 | 83.95 | *S. suis* |
| A3 | SS34 | JBZGLI000000000 | 99.96 | 83.82 | *S. suis* |
| A5 | SS35 | JBZGLH000000000 | 99.87 | 83.91 | *S. suis* |
| A7 | SS36 | JBZGLG000000000 | 99.97 | 83.89 | *S. suis* |
| A9 | SS37 | JBZGLF000000000 | 99.96 | 83.63 | *S. suis* |
| B3 | SS38 | JBZGLE000000000 | 99.95 | 83.89 | *S. suis* |
| B5 | SS39 | JBZGLD000000000 | 99.90 | 83.92 | *S. suis* |
| B7 | SS40 | JBZGLC000000000 | 99.99 | 83.85 | *S. suis* |
| B9 | SS41 | JBZGLB000000000 | 96.81 | 83.77 | *S. suis* |

**Abbreviations:** ANI, average nucleotide identity. ANI was calculated using the BLAST-based ANIb method implemented in JSpeciesWS. *Streptococcus suis* P1/7 and *Streptococcus parasuis* SUT-286 were used as reference genomes. Isolate IDs represent laboratory strain codes, and sample IDs correspond to the records deposited under NCBI BioProject PRJNA1439339.

Supplementary Table S3. Epidemiological and molecular characteristics of individual isolates

| Isolate ID | Year | Specimen source | Serotype | ST | Sex | Age |
| --- | --- | --- | --- | --- | --- | --- |
| 4 | 2015 | Cerebrospinal fluid | 2 | 1 | Male | 53 |
| 6 | 2015 | Cerebrospinal fluid | 2 | 1 | Male | 64 |
| 7 | 2015 | Cerebrospinal fluid | 2 | 1 | Female | 73 |
| 11 | 2016 | Blood | 2 | 1 | Male | 63 |
| 12 | 2016 | Cerebrospinal fluid | 2 | 1 | Male | 43 |
| 13 | 2016 | Cerebrospinal fluid | 2 | 1 | Male | 40 |
| 14 | 2016 | Blood | 2 | 1 | Male | 71 |
| 15 | 2016 | Cerebrospinal fluid | 2 | 1 | Male | 63 |
| 16 | 2016 | Cerebrospinal fluid | 2 | 1 | Male | 62 |
| 18 | 2016 | Cerebrospinal fluid | 2 | 1 | Male | 53 |
| 19 | 2016 | Cerebrospinal fluid | 2 | 1005 | Male | 63 |
| 20 | 2016 | Cerebrospinal fluid | 14 | 7 | Male | 53 |
| 21 | 2016 | Cerebrospinal fluid | 2 | 1 | Male | 43 |
| 22 | 2016 | Cerebrospinal fluid | 2 | 1 | Male | 23 |
| 23 | 2017 | Cerebrospinal fluid | 2 | 1 | Male | 55 |
| 24 | 2017 | Cerebrospinal fluid | 14 | 7 | Male | 59 |
| 25 | 2018 | Cerebrospinal fluid | 2 | 1 | Male | 89 |
| 26 | 2018 | Blood | NA | NA | Female | 27 |
| D7 | 2018 | Blood | NA | NA | Male | 53 |
| C3 | 2019 | Cerebrospinal fluid | 14 | 7 | Male | 55 |
| E5 | 2019 | Cerebrospinal fluid | 14 | 1 | Male | 46 |
| E3 | 2019 | Blood | 2 | 1 | Female | 75 |
| D9 | 2019 | Blood | 2 | 1 | Male | 47 |
| E1 | 2019 | Blood | 2 | 1 | Male | 61 |
| C1 | 2019 | Blood | 2 | 7 | Male | 56 |
| C5 | 2020 | Cerebrospinal fluid | 2 | 1 | Male | 44 |
| C7 | 2020 | Cerebrospinal fluid | 14 | 7 | Male | 68 |
| C9 | 2020 | Cerebrospinal fluid | 14 | 7 | Male | 67 |
| B1 | 2020 | Cerebrospinal fluid | 2 | 1 | Male | 57 |
| D1 | 2020 | Blood | 2 | 1 | Male | 73 |
| D3 | 2020 | Blood | 2 | 1 | Male | 42 |
| D5 | 2020 | Blood | 2 | 1 | Male | 68 |
| A1 | 2020 | Cerebrospinal fluid | 2 | 1 | Female | 66 |
| A3 | 2021 | Blood | 2 | 1 | Male | 54 |
| A5 | 2021 | Cerebrospinal fluid | 14 | 1 | Male | 37 |
| A7 | 2021 | Blood | 2 | 1 | Male | 52 |
| A9 | 2021 | Blood | 2 | 956 | Male | 39 |
| B3 | 2021 | Cerebrospinal fluid | 2 | 1 | Male | 47 |
| B5 | 2021 | Blood | 2 | 7 | Male | 53 |
| B7 | 2021 | Blood | 2 | 1 | Male | 48 |
| B9 | 2021 | Cerebrospinal fluid | 2 | 25 | Male | 39 |

Abbreviations: ST, sequence type; NA, not assigned. Isolates 26 and D7 had unresolved species assignments and were excluded from the final *S. suis* dataset. Serotypes and sequence types were not assigned using the *S. suis* schemes.

Supplementary Table S4. Isolate-level comparison of antimicrobial resistance genotypes and phenotypes

| Isolate | CLI MIC | CLI phenotype | Lincosamide-associated genes | ERY MIC | ERY phenotype | Macrolide-associated genes | TET MIC | TET phenotype | TET-associated genes |
| --- | --- | --- | --- | --- | --- | --- | --- | --- | --- |
| 4 | 0.25 | S | None detected | 0.25 | S | None detected | 16 | R | *tet(M)* |
| 6 | 0.25 | S | None detected | 0.25 | S | None detected | 32 | R | *tet(M)* |
| 7 | 0.25 | S | None detected | 0.25 | S | None detected | 32 | R | *tet(M)* |
| 11 | 0.25 | S | None detected | 0.25 | S | None detected | 32 | R | *tet(M)* |
| 12 | 0.25 | S | None detected | 0.25 | S | None detected | 32 | R | *tet(O)* |
| 13 | 4 | R | None detected | 4 | R | None detected | 32 | R | *tet(M)* |
| 14 | 8 | R | *erm(B)* | 4 | R | *erm(B)* | 16 | R | *tet(M)* |
| 15 | 4 | R | *erm(B), lnu(B), lsa(E)* | 8 | R | *erm(B)* | 32 | R | *tet(M)* |
| 16 | 0.25 | S | None detected | 0.25 | S | None detected | 32 | R | *tet(M)* |
| 18 | 4 | R | *erm(B)* | 4 | R | *erm(B)* | 16 | R | *tet(O)* |
| 19 | 4 | R | *erm(B)* | 4 | R | *erm(B)* | 16 | R | *tet(M)* |
| 20 | 4 | R | *erm(B)* | 4 | R | *erm(B)* | 32 | R | *tet(40), tet(O)* |
| 21 | 0.25 | S | None detected | 0.25 | S | None detected | 8 | R | *tet(M)* |
| 22 | 4 | R | *erm(B)* | 8 | R | *erm(B)* | 32 | R | *tet(O), tet(M)* |
| 23 | 4 | R | *erm(B)* | 8 | R | *erm(B)* | 32 | R | *tet(M), tet(W)* |
| 24 | 4 | R | None detected | 4 | R | None detected | 32 | R | *tet(40), tet(O)* |
| 25 | 4 | R | *erm(B)* | 4 | R | *erm(B)* | 32 | R | None detected |
| C3 | 4 | R | *erm(B)* | 8 | R | *erm(B)* | 32 | R | *tet(40), tet(O)* |
| E5 | 0.25 | S | None detected | 0.25 | S | None detected | 16 | R | *tet(M)* |
| E3 | 4 | R | *erm(B)* | 8 | R | *erm(B)* | 32 | R | *tet(M), tet(L)* |
| D9 | 4 | R | *erm(B)* | 8 | R | *erm(B)* | 8 | R | *tet(O)* |
| E1 | 0.25 | S | None detected | 0.25 | S | None detected | 16 | R | *tet(M)* |
| C1 | 4 | R | *erm(B)* | 8 | R | *msr(D), mef(A), erm(B)* | 32 | R | *tet(40), tet(O)* |
| C5 | 0.25 | S | None detected | 0.25 | S | None detected | 2 | S | None detected |
| C7 | 4 | R | *erm(B), lnu(C)* | 8 | R | *erm(B)* | 32 | R | *tet(40), tet(O/W/32/O)* |
| C9 | 4 | R | *erm(B)* | 8 | R | *erm(B)* | 32 | R | *tet(40), tet(O)* |
| B1 | 0.25 | S | None detected | 0.25 | S | None detected | 32 | R | *tet(M)* |
| D1 | 0.25 | S | None detected | 0.25 | S | None detected | 16 | R | *tet(O)* |
| D3 | 4 | R | *erm(B), lnu(E)* | 1 | R | *erm(B)* | 32 | R | *tet(M)* |
| D5 | 4 | R | *erm(B)* | 8 | R | *erm(B)* | 32 | R | *tet(O)* |
| A1 | 0.25 | S | None detected | 0.25 | S | None detected | 32 | R | *tet(M)* |
| A3 | 4 | R | *erm(B)* | 8 | R | *erm(B)* | 32 | R | *tet(O)* |
| A5 | 4 | R | *erm(B)* | 8 | R | *erm(B)* | 32 | R | *tet(O)* |
| A7 | 4 | R | *erm(B)* | 8 | R | *erm(B)* | 32 | R | *tet(M), tet(L)* |
| A9 | 4 | R | *erm(B)* | 8 | R | *erm(B)* | 32 | R | *tet(O)* |
| B3 | 4 | R | *erm(B)* | 4 | R | *erm(B)* | 32 | R | *tet(O)* |
| B5 | 4 | R | *erm(B)* | 8 | R | *erm(B)* | 32 | R | *tet(40), tet(O)* |
| B7 | 4 | R | None detected | 4 | R | None detected | 16 | R | *tet(O)* |
| B9 | 4 | R | *erm(B)* | 8 | R | *erm(B)* | 32 | R | *tet(O)* |

Abbreviations: CLI, clindamycin; ERY, erythromycin; TET, tetracycline; MIC, minimum inhibitory concentration; S, susceptible; R, resistant. Resistance-associated genes were identified using ResFinder with thresholds of >95% coverage and >99% sequence identity. The table presents the isolate-level relationship between antimicrobial susceptibility phenotypes and the corresponding resistance-associated genes. “None detected” indicates that no corresponding acquired resistance gene meeting the predefined thresholds was identified.

Supplementary Table S5. Isolate-level MIC values and interpretations for the 39 genome-confirmed Streptococcus suis isolates

| Isolate ID | AMP MIC | AMP result | CRO MIC | CRO result | CLI MIC | CLI result | ERY MIC | ERY result | LVX MIC | LVX result | LZD MIC | LZD result | TET MIC | TET result | PEN MIC | PEN result | VAN MIC | VAN result | MEM MIC | MEM result |
| --- | --- | --- | --- | --- | --- | --- | --- | --- | --- | --- | --- | --- | --- | --- | --- | --- | --- | --- | --- | --- |
| 4 | 0.25 | S | 0.5 | S | 0.25 | S | 0.25 | S | 0.25 | S | 2 | S | 16 | R | 2 | I | 1 | S | 0.25 | S |
| 6 | 0.25 | S | 0.5 | S | 0.25 | S | 0.25 | S | 2 | S | 2 | S | 32 | R | 2 | I | 1 | S | 0.25 | S |
| 7 | 0.25 | S | 0.5 | S | 0.25 | S | 0.25 | S | 2 | S | 2 | S | 32 | R | 2 | I | 1 | S | 0.25 | S |
| 11 | 0.25 | S | 0.5 | S | 0.25 | S | 0.25 | S | 2 | S | 2 | S | 32 | R | 0.06 | S | 1 | S | 0.25 | S |
| 12 | 0.25 | S | 0.5 | S | 0.25 | S | 0.25 | S | 2 | S | 2 | S | 32 | R | 0.06 | S | 1 | S | 0.25 | S |
| 13 | 0.25 | S | 0.5 | S | 4 | R | 4 | R | 2 | S | 2 | S | 32 | R | 0.06 | S | 1 | S | 0.25 | S |
| 14 | 0.25 | S | 0.5 | S | 8 | R | 4 | R | 0.25 | S | 2 | S | 16 | R | 0.12 | S | 1 | S | 0.25 | S |
| 15 | 0.25 | S | 0.5 | S | 4 | R | 8 | R | 2 | S | 2 | S | 32 | R | 0.12 | S | 1 | S | 0.25 | S |
| 16 | 0.25 | S | 0.5 | S | 0.25 | S | 0.25 | S | 0.25 | S | 2 | S | 32 | R | 0.06 | S | 1 | S | 0.25 | S |
| 18 | 0.25 | S | 0.5 | S | 4 | R | 4 | R | 2 | S | 2 | S | 16 | R | 0.12 | S | 1 | S | 0.25 | S |
| 19 | 0.25 | S | 0.5 | S | 4 | R | 4 | R | 2 | S | 2 | S | 16 | R | 0.12 | S | 1 | S | 0.25 | S |
| 20 | 0.25 | S | 0.5 | S | 4 | R | 4 | R | 2 | S | 2 | S | 32 | R | 0.12 | S | 1 | S | 0.25 | S |
| 21 | 0.25 | S | 0.5 | S | 0.25 | S | 0.25 | S | 2 | S | 2 | S | 8 | R | 0.12 | S | 1 | S | 0.25 | S |
| 22 | 0.25 | S | 0.5 | S | 4 | R | 8 | R | 2 | S | 2 | S | 32 | R | 2 | I | 1 | S | 0.25 | S |
| 23 | 0.25 | S | 0.5 | S | 4 | R | 8 | R | 0.25 | S | 2 | S | 32 | R | 0.12 | S | 1 | S | 0.25 | S |
| 24 | 0.25 | S | 0.5 | S | 4 | R | 4 | R | 0.25 | S | 2 | S | 32 | R | 0.06 | S | 1 | S | 0.25 | S |
| 25 | 0.25 | S | 0.5 | S | 4 | R | 4 | R | 2 | S | 2 | S | 32 | R | 0.06 | S | 1 | S | 0.25 | S |
| C3 | 0.25 | S | 0.5 | S | 4 | R | 8 | R | 2 | S | 2 | S | 32 | R | 0.06 | S | 1 | S | 0.25 | S |
| E5 | 0.25 | S | 0.5 | S | 0.25 | S | 0.25 | S | 2 | S | 2 | S | 16 | R | 0.06 | S | 1 | S | 0.25 | S |
| E3 | 0.25 | S | 0.5 | S | 4 | R | 8 | R | 2 | S | 2 | S | 32 | R | 0.06 | S | 1 | S | 0.25 | S |
| D9 | 0.25 | S | 0.5 | S | 4 | R | 8 | R | 2 | S | 2 | S | 8 | R | 0.06 | S | 1 | S | 0.25 | S |
| E1 | 0.25 | S | 0.5 | S | 0.25 | S | 0.25 | S | 2 | S | 2 | S | 16 | R | 0.06 | S | 1 | S | 0.25 | S |
| C1 | 0.25 | S | 0.5 | S | 4 | R | 8 | R | 2 | S | 2 | S | 32 | R | 0.12 | S | 1 | S | 0.25 | S |
| C5 | 0.25 | S | 0.5 | S | 0.25 | S | 0.25 | S | 2 | S | 2 | S | 2 | S | 0.06 | S | 1 | S | 0.25 | S |
| C7 | 0.25 | S | 0.5 | S | 4 | R | 8 | R | 2 | S | 2 | S | 32 | R | 0.06 | S | 1 | S | 0.25 | S |
| C9 | 0.25 | S | 0.5 | S | 4 | R | 8 | R | 2 | S | 2 | S | 32 | R | 0.06 | S | 1 | S | 0.25 | S |
| B1 | 0.25 | S | 0.5 | S | 0.25 | S | 0.25 | S | 2 | S | 2 | S | 32 | R | 0.06 | S | 1 | S | 0.25 | S |
| D1 | 0.25 | S | 0.5 | S | 0.25 | S | 0.25 | S | 2 | S | 2 | S | 16 | R | 0.06 | S | 1 | S | 0.25 | S |
| D3 | 0.25 | S | 0.5 | S | 4 | R | 1 | R | 2 | S | 2 | S | 32 | R | 0.06 | S | 1 | S | 0.25 | S |
| D5 | 0.25 | S | 0.5 | S | 4 | R | 8 | R | 2 | S | 2 | S | 32 | R | 0.06 | S | 1 | S | 0.25 | S |
| A1 | 0.25 | S | 0.5 | S | 0.25 | S | 0.25 | S | 2 | S | 2 | S | 32 | R | 0.06 | S | 1 | S | 0.25 | S |
| A3 | 0.25 | S | 0.5 | S | 4 | R | 8 | R | 2 | S | 2 | S | 32 | R | 0.06 | S | 1 | S | 0.25 | S |
| A5 | 0.25 | S | 0.5 | S | 4 | R | 8 | R | 2 | S | 2 | S | 32 | R | 0.06 | S | 1 | S | 0.25 | S |
| A7 | 0.25 | S | 0.5 | S | 4 | R | 8 | R | 2 | S | 2 | S | 32 | R | 0.06 | S | 1 | S | 0.25 | S |
| A9 | 0.25 | S | 0.5 | S | 4 | R | 8 | R | 2 | S | 2 | S | 32 | R | 0.06 | S | 1 | S | 0.25 | S |
| B3 | 0.25 | S | 0.5 | S | 4 | R | 4 | R | 2 | S | 2 | S | 32 | R | 0.06 | S | 1 | S | 0.25 | S |
| B5 | 0.25 | S | 0.5 | S | 4 | R | 8 | R | 2 | S | 2 | S | 32 | R | 0.06 | S | 1 | S | 0.25 | S |
| B7 | 0.25 | S | 0.5 | S | 4 | R | 4 | R | 2 | S | 2 | S | 16 | R | 0.06 | S | 1 | S | 0.25 | S |
| B9 | 0.25 | S | 0.5 | S | 4 | R | 8 | R | 2 | S | 2 | S | 32 | R | 0.06 | S | 1 | S | 0.25 | S |

Notes: MIC values are expressed in μg/mL. S, susceptible; I, intermediate; R, resistant.

Abbreviations: AMP, ampicillin; CRO, ceftriaxone; CLI, clindamycin; ERY, erythromycin; LVX, levofloxacin; LZD, linezolid; TET, tetracycline; PEN, penicillin; VAN, vancomycin; MEM, meropenem. MIC breakpoints are expressed in μg/mL.

Supplementary Table S6. MIC interpretive breakpoints used in this study

| Antimicrobial agent | CLSI organism group used for interpretation | Susceptible | Intermediate | Resistant |
| --- | --- | --- | --- | --- |
| Ampicillin | *Streptococcus* spp*. viridans group* | ≤0.25 | 0.5-4 | ≥8 |
| Ceftriaxone | *Streptococcus* spp. viridans group | ≤1 | 2.0 | ≥4 |
| Clindamycin | *Streptococcus* spp. viridans group | ≤0.25 | 0.5 | ≥1 |
| Erythromycin | *Streptococcus* spp. viridans group | ≤0.25 | 0.5 | ≥1 |
| Levofloxacin | *Streptococcus* spp. viridans group | ≤2 | 4.0 | ≥8 |
| Linezolid | *Streptococcus* spp. viridans group | ≤2 | - | - |
| Tetracycline | *Streptococcus* spp. viridans group | ≤2 | 4.0 | ≥8 |
| Penicillin | *Streptococcus* spp. viridans group | ≤0.12 | 0.25-2 | ≥4 |
| Vancomycin | *Streptococcus* spp. viridans group | ≤1 | - | - |
| Meropenem | *Streptococcus* spp. viridans group | ≤0.25 | - | - |

Notes: A dash indicates that no intermediate or resistant category was provided in the source table.

Supplementary Table S7. Comparison of virulence-associated and antimicrobial resistance gene carriage between the major serotypes and sequence types of *S. suis* isolates

| Gene | Gene category | Functional/antimicrobial class | Serotype 2, n/N (%) | Serotype 14, n/N (%) | *P* (serotype) | FDR-adjusted *P* (serotype) | ST1, n/N (%) | ST7, n/N (%) | *P* (ST) | FDR-adjusted *P* (ST) |
| --- | --- | --- | --- | --- | --- | --- | --- | --- | --- | --- |
| *GBSSAG0907_homologue* | Virulence-associated | Others | 31/32 (96.9%) | 7/7 (100.0%) | 1.000 | 1.000 | 29/29 (100.0%) | 7/7 (100.0%) | 1.000 | 1.000 |
| *SMU61_homologue* | Virulence-associated | Others | 31/32 (96.9%) | 7/7 (100.0%) | 1.000 | 1.000 | 29/29 (100.0%) | 7/7 (100.0%) | 1.000 | 1.000 |
| *adcR* | Virulence-associated | Transcriptional factors/TCS | 32/32 (100.0%) | 7/7 (100.0%) | 1.000 | 1.000 | 29/29 (100.0%) | 7/7 (100.0%) | 1.000 | 1.000 |
| *adhesinP* | Virulence-associated | Others | 30/32 (93.8%) | 7/7 (100.0%) | 1.000 | 1.000 | 28/29 (96.6%) | 7/7 (100.0%) | 1.000 | 1.000 |
| *amylopullulanase* | Virulence-associated | Others | 32/32 (100.0%) | 7/7 (100.0%) | 1.000 | 1.000 | 29/29 (100.0%) | 7/7 (100.0%) | 1.000 | 1.000 |
| *aroA* | Virulence-associated | Enzymes/proteases | 32/32 (100.0%) | 7/7 (100.0%) | 1.000 | 1.000 | 29/29 (100.0%) | 7/7 (100.0%) | 1.000 | 1.000 |
| *ccpA* | Virulence-associated | Transcriptional factors/TCS | 32/32 (100.0%) | 7/7 (100.0%) | 1.000 | 1.000 | 29/29 (100.0%) | 7/7 (100.0%) | 1.000 | 1.000 |
| *cdd* | Virulence-associated | Enzymes/proteases | 32/32 (100.0%) | 7/7 (100.0%) | 1.000 | 1.000 | 29/29 (100.0%) | 7/7 (100.0%) | 1.000 | 1.000 |
| *ciaH* | Virulence-associated | Transcriptional factors/TCS | 32/32 (100.0%) | 7/7 (100.0%) | 1.000 | 1.000 | 29/29 (100.0%) | 7/7 (100.0%) | 1.000 | 1.000 |
| *ciaR* | Virulence-associated | Transcriptional factors/TCS | 32/32 (100.0%) | 7/7 (100.0%) | 1.000 | 1.000 | 29/29 (100.0%) | 7/7 (100.0%) | 1.000 | 1.000 |
| *dltA* | Virulence-associated | Enzymes/proteases | 32/32 (100.0%) | 7/7 (100.0%) | 1.000 | 1.000 | 29/29 (100.0%) | 7/7 (100.0%) | 1.000 | 1.000 |
| *dpr* | Virulence-associated | Stress survival | 32/32 (100.0%) | 7/7 (100.0%) | 1.000 | 1.000 | 29/29 (100.0%) | 7/7 (100.0%) | 1.000 | 1.000 |
| *endoD* | Virulence-associated | Enzymes/proteases | 31/32 (96.9%) | 7/7 (100.0%) | 1.000 | 1.000 | 29/29 (100.0%) | 7/7 (100.0%) | 1.000 | 1.000 |
| *eno* | Virulence-associated | Enzymes/proteases | 32/32 (100.0%) | 7/7 (100.0%) | 1.000 | 1.000 | 29/29 (100.0%) | 7/7 (100.0%) | 1.000 | 1.000 |
| *epf* | Virulence-associated | Surface/secreted components | 31/32 (96.9%) | 7/7 (100.0%) | 1.000 | 1.000 | 29/29 (100.0%) | 7/7 (100.0%) | 1.000 | 1.000 |
| *fbpS* | Virulence-associated | Surface/secreted components | 32/32 (100.0%) | 7/7 (100.0%) | 1.000 | 1.000 | 29/29 (100.0%) | 7/7 (100.0%) | 1.000 | 1.000 |
| *feoB* | Virulence-associated | Others | 32/32 (100.0%) | 7/7 (100.0%) | 1.000 | 1.000 | 29/29 (100.0%) | 7/7 (100.0%) | 1.000 | 1.000 |
| *fur* | Virulence-associated | Regulation | 32/32 (100.0%) | 7/7 (100.0%) | 1.000 | 1.000 | 29/29 (100.0%) | 7/7 (100.0%) | 1.000 | 1.000 |
| *gdh* | Virulence-associated | Enzymes/proteases | 32/32 (100.0%) | 7/7 (100.0%) | 1.000 | 1.000 | 29/29 (100.0%) | 7/7 (100.0%) | 1.000 | 1.000 |
| *glnA* | Virulence-associated | Enzymes/proteases | 31/32 (96.9%) | 7/7 (100.0%) | 1.000 | 1.000 | 29/29 (100.0%) | 7/7 (100.0%) | 1.000 | 1.000 |
| *glnH* | Virulence-associated | Others | 32/32 (100.0%) | 7/7 (100.0%) | 1.000 | 1.000 | 29/29 (100.0%) | 7/7 (100.0%) | 1.000 | 1.000 |
| *gnd* | Virulence-associated | Others | 32/32 (100.0%) | 7/7 (100.0%) | 1.000 | 1.000 | 29/29 (100.0%) | 7/7 (100.0%) | 1.000 | 1.000 |
| *gpmA* | Virulence-associated | Others | 32/32 (100.0%) | 7/7 (100.0%) | 1.000 | 1.000 | 29/29 (100.0%) | 7/7 (100.0%) | 1.000 | 1.000 |
| *gtfA* | Virulence-associated | Enzymes/proteases | 32/32 (100.0%) | 7/7 (100.0%) | 1.000 | 1.000 | 29/29 (100.0%) | 7/7 (100.0%) | 1.000 | 1.000 |
| *guaA* | Virulence-associated | Nutritional/Metabolic factor | 32/32 (100.0%) | 7/7 (100.0%) | 1.000 | 1.000 | 29/29 (100.0%) | 7/7 (100.0%) | 1.000 | 1.000 |
| *guaB* | Virulence-associated | Nutritional/Metabolic factor | 32/32 (100.0%) | 7/7 (100.0%) | 1.000 | 1.000 | 29/29 (100.0%) | 7/7 (100.0%) | 1.000 | 1.000 |
| *htpS* | Virulence-associated | Surface/secreted components | 32/32 (100.0%) | 6/7 (85.7%) | 0.179 | 1.000 | 28/29 (96.6%) | 7/7 (100.0%) | 1.000 | 1.000 |
| *hylA* | Virulence-associated | Others | 32/32 (100.0%) | 7/7 (100.0%) | 1.000 | 1.000 | 29/29 (100.0%) | 7/7 (100.0%) | 1.000 | 1.000 |
| *lgt* | Virulence-associated | Post-translational modification | 32/32 (100.0%) | 7/7 (100.0%) | 1.000 | 1.000 | 29/29 (100.0%) | 7/7 (100.0%) | 1.000 | 1.000 |
| *lmb* | Virulence-associated | Adherence | 32/32 (100.0%) | 7/7 (100.0%) | 1.000 | 1.000 | 29/29 (100.0%) | 7/7 (100.0%) | 1.000 | 1.000 |
| *lpp* | Virulence-associated | Others | 32/32 (100.0%) | 7/7 (100.0%) | 1.000 | 1.000 | 29/29 (100.0%) | 7/7 (100.0%) | 1.000 | 1.000 |
| *lspA* | Virulence-associated | Post-translational modification | 32/32 (100.0%) | 7/7 (100.0%) | 1.000 | 1.000 | 29/29 (100.0%) | 7/7 (100.0%) | 1.000 | 1.000 |
| *luxS* | Virulence-associated | Enzymes/proteases | 32/32 (100.0%) | 7/7 (100.0%) | 1.000 | 1.000 | 29/29 (100.0%) | 7/7 (100.0%) | 1.000 | 1.000 |
| *manN* | Virulence-associated | Others | 32/32 (100.0%) | 7/7 (100.0%) | 1.000 | 1.000 | 29/29 (100.0%) | 7/7 (100.0%) | 1.000 | 1.000 |
| *mrp* | Virulence-associated | Surface/secreted components | 29/32 (90.6%) | 2/7 (28.6%) | 0.002 | 0.122 | 29/29 (100.0%) | 0/7 (0.0%) | < 0.001 | < 0.001 |
| *nadR* | Virulence-associated | Transcriptional factors/TCS | 31/32 (96.9%) | 7/7 (100.0%) | 1.000 | 1.000 | 29/29 (100.0%) | 7/7 (100.0%) | 1.000 | 1.000 |
| *neuB* | Virulence-associated | Enzymes/proteases | 32/32 (100.0%) | 7/7 (100.0%) | 1.000 | 1.000 | 29/29 (100.0%) | 7/7 (100.0%) | 1.000 | 1.000 |
| *ofs* | Virulence-associated | Surface/secreted components | 31/32 (96.9%) | 7/7 (100.0%) | 1.000 | 1.000 | 29/29 (100.0%) | 7/7 (100.0%) | 1.000 | 1.000 |
| *orf207* | Virulence-associated | Others | 32/32 (100.0%) | 7/7 (100.0%) | 1.000 | 1.000 | 29/29 (100.0%) | 7/7 (100.0%) | 1.000 | 1.000 |
| *pepXP* | Virulence-associated | Others | 32/32 (100.0%) | 7/7 (100.0%) | 1.000 | 1.000 | 29/29 (100.0%) | 7/7 (100.0%) | 1.000 | 1.000 |
| *permease* | Virulence-associated | Others | 32/32 (100.0%) | 7/7 (100.0%) | 1.000 | 1.000 | 29/29 (100.0%) | 7/7 (100.0%) | 1.000 | 1.000 |
| *pgdA* | Virulence-associated | Enzymes/proteases | 32/32 (100.0%) | 7/7 (100.0%) | 1.000 | 1.000 | 29/29 (100.0%) | 7/7 (100.0%) | 1.000 | 1.000 |
| *phospholipaseC* | Virulence-associated | Others | 32/32 (100.0%) | 7/7 (100.0%) | 1.000 | 1.000 | 29/29 (100.0%) | 7/7 (100.0%) | 1.000 | 1.000 |
| *pilT_gapA* | Virulence-associated | Others | 32/32 (100.0%) | 7/7 (100.0%) | 1.000 | 1.000 | 29/29 (100.0%) | 7/7 (100.0%) | 1.000 | 1.000 |
| *purA* | Virulence-associated | Enzymes/proteases | 32/32 (100.0%) | 7/7 (100.0%) | 1.000 | 1.000 | 29/29 (100.0%) | 7/7 (100.0%) | 1.000 | 1.000 |
| *purD* | Virulence-associated | Enzymes/proteases | 32/32 (100.0%) | 7/7 (100.0%) | 1.000 | 1.000 | 29/29 (100.0%) | 7/7 (100.0%) | 1.000 | 1.000 |
| *revS* | Virulence-associated | Transcriptional factors/TCS | 31/32 (96.9%) | 7/7 (100.0%) | 1.000 | 1.000 | 29/29 (100.0%) | 7/7 (100.0%) | 1.000 | 1.000 |
| *rgg* | Virulence-associated | Transcriptional factors/TCS | 32/32 (100.0%) | 7/7 (100.0%) | 1.000 | 1.000 | 29/29 (100.0%) | 7/7 (100.0%) | 1.000 | 1.000 |
| *salK* | Virulence-associated | Transcriptional factors/TCS | 17/32 (53.1%) | 1/7 (14.3%) | 0.098 | 1.000 | 17/29 (58.6%) | 0/7 (0.0%) | 0.008 | 0.195 |
| *salR* | Virulence-associated | Transcriptional factors/TCS | 19/32 (59.4%) | 1/7 (14.3%) | 0.044 | 1.000 | 19/29 (65.5%) | 0/7 (0.0%) | 0.002 | 0.082 |
| *sao* | Virulence-associated | Surface/secreted components | 32/32 (100.0%) | 7/7 (100.0%) | 1.000 | 1.000 | 29/29 (100.0%) | 7/7 (100.0%) | 1.000 | 1.000 |
| *scrB* | Virulence-associated | Enzymes/proteases | 32/32 (100.0%) | 7/7 (100.0%) | 1.000 | 1.000 | 29/29 (100.0%) | 7/7 (100.0%) | 1.000 | 1.000 |
| *scrR* | Virulence-associated | Transcriptional factors/TCS | 32/32 (100.0%) | 7/7 (100.0%) | 1.000 | 1.000 | 29/29 (100.0%) | 7/7 (100.0%) | 1.000 | 1.000 |
| *sly* | Virulence-associated | Surface/secreted components | 31/32 (96.9%) | 7/7 (100.0%) | 1.000 | 1.000 | 29/29 (100.0%) | 7/7 (100.0%) | 1.000 | 1.000 |
| *sodA* | Virulence-associated | Stress survival | 32/32 (100.0%) | 7/7 (100.0%) | 1.000 | 1.000 | 29/29 (100.0%) | 7/7 (100.0%) | 1.000 | 1.000 |
| *srtA* | Virulence-associated | Enzymes/proteases | 32/32 (100.0%) | 7/7 (100.0%) | 1.000 | 1.000 | 29/29 (100.0%) | 7/7 (100.0%) | 1.000 | 1.000 |
| *srtF* | Virulence-associated | Others | 32/32 (100.0%) | 7/7 (100.0%) | 1.000 | 1.000 | 29/29 (100.0%) | 7/7 (100.0%) | 1.000 | 1.000 |
| *srtF-sbp1* | Virulence-associated | Others | 32/32 (100.0%) | 7/7 (100.0%) | 1.000 | 1.000 | 29/29 (100.0%) | 7/7 (100.0%) | 1.000 | 1.000 |
| *srtF-sbpF* | Virulence-associated | Others | 31/32 (96.9%) | 7/7 (100.0%) | 1.000 | 1.000 | 29/29 (100.0%) | 7/7 (100.0%) | 1.000 | 1.000 |
| *srtG* | Virulence-associated | Adherence | 1/32 (3.1%) | 0/7 (0.0%) | 1.000 | 1.000 | 0/29 (0.0%) | 0/7 (0.0%) | 1.000 | 1.000 |
| *srtG-sgp1* | Virulence-associated | Adherence | 1/32 (3.1%) | 0/7 (0.0%) | 1.000 | 1.000 | 0/29 (0.0%) | 0/7 (0.0%) | 1.000 | 1.000 |
| *srtG-sgp2* | Virulence-associated | Adherence | 1/32 (3.1%) | 0/7 (0.0%) | 1.000 | 1.000 | 0/29 (0.0%) | 0/7 (0.0%) | 1.000 | 1.000 |
| *ssnA* | Virulence-associated | Enzymes/proteases | 32/32 (100.0%) | 7/7 (100.0%) | 1.000 | 1.000 | 29/29 (100.0%) | 7/7 (100.0%) | 1.000 | 1.000 |
| *sspA* | Virulence-associated | Surface/secreted components | 32/32 (100.0%) | 7/7 (100.0%) | 1.000 | 1.000 | 29/29 (100.0%) | 7/7 (100.0%) | 1.000 | 1.000 |
| *traG* | Virulence-associated | Surface/secreted components | 31/32 (96.9%) | 6/7 (85.7%) | 0.331 | 1.000 | 28/29 (96.6%) | 6/7 (85.7%) | 0.356 | 1.000 |
| *treR* | Virulence-associated | Transcriptional factors/TCS | 32/32 (100.0%) | 7/7 (100.0%) | 1.000 | 1.000 | 29/29 (100.0%) | 7/7 (100.0%) | 1.000 | 1.000 |
| *troA* | Virulence-associated | Nutritional/Metabolic factor | 32/32 (100.0%) | 7/7 (100.0%) | 1.000 | 1.000 | 29/29 (100.0%) | 7/7 (100.0%) | 1.000 | 1.000 |
| *vicR-covR* | Virulence-associated | Transcriptional factors/TCS | 32/32 (100.0%) | 7/7 (100.0%) | 1.000 | 1.000 | 29/29 (100.0%) | 7/7 (100.0%) | 1.000 | 1.000 |
| *virA* | Virulence-associated | Others | 32/32 (100.0%) | 7/7 (100.0%) | 1.000 | 1.000 | 29/29 (100.0%) | 7/7 (100.0%) | 1.000 | 1.000 |
| *zmpC* | Virulence-associated | Immune modulation | 32/32 (100.0%) | 7/7 (100.0%) | 1.000 | 1.000 | 29/29 (100.0%) | 7/7 (100.0%) | 1.000 | 1.000 |
| *tet(40)* | Antimicrobial resistance | Tetracyclines | 2/32 (6.2%) | 5/7 (71.4%) | 0.001 | 0.013 | 0/29 (0.0%) | 7/7 (100.0%) | < 0.001 | < 0.001 |
| *tet(O)* | Antimicrobial resistance | Tetracyclines | 13/32 (40.6%) | 5/7 (71.4%) | 0.215 | 0.682 | 10/29 (34.5%) | 6/7 (85.7%) | 0.030 | 0.189 |
| *tet(M)* | Antimicrobial resistance | Tetracyclines | 18/32 (56.2%) | 1/7 (14.3%) | 0.091 | 0.682 | 18/29 (62.1%) | 0/7 (0.0%) | 0.008 | 0.072 |
| *tet(L)* | Antimicrobial resistance | Tetracyclines | 2/32 (6.2%) | 0/7 (0.0%) | 1.000 | 1.000 | 2/29 (6.9%) | 0/7 (0.0%) | 1.000 | 1.000 |
| *tet(W)* | Antimicrobial resistance | Tetracyclines | 1/32 (3.1%) | 0/7 (0.0%) | 1.000 | 1.000 | 1/29 (3.4%) | 0/7 (0.0%) | 1.000 | 1.000 |
| *tet(O/W/32/O)* | Antimicrobial resistance | Tetracyclines | 0/32 (0.0%) | 1/7 (14.3%) | 0.179 | 0.682 | 0/29 (0.0%) | 1/7 (14.3%) | 0.194 | 0.369 |
| *ant(6)-Ia* | Antimicrobial resistance | Aminoglycosides | 20/32 (62.5%) | 5/7 (71.4%) | 1.000 | 1.000 | 19/29 (65.5%) | 5/7 (71.4%) | 1.000 | 1.000 |
| *aph(3′)-III* | Antimicrobial resistance | Aminoglycosides | 5/32 (15.6%) | 1/7 (14.3%) | 1.000 | 1.000 | 5/29 (17.2%) | 1/7 (14.3%) | 1.000 | 1.000 |
| *aac(6′)-aph(2″)* | Antimicrobial resistance | Aminoglycosides | 2/32 (6.2%) | 2/7 (28.6%) | 0.141 | 0.682 | 2/29 (6.9%) | 2/7 (28.6%) | 0.163 | 0.369 |
| *msr(D)* | Antimicrobial resistance | Macrolides | 1/32 (3.1%) | 0/7 (0.0%) | 1.000 | 1.000 | 0/29 (0.0%) | 1/7 (14.3%) | 0.194 | 0.369 |
| *mef(A)* | Antimicrobial resistance | Macrolides | 1/32 (3.1%) | 0/7 (0.0%) | 1.000 | 1.000 | 0/29 (0.0%) | 1/7 (14.3%) | 0.194 | 0.369 |
| *erm(B)* | Antimicrobial resistance | Macrolides | 18/32 (56.2%) | 5/7 (71.4%) | 0.678 | 1.000 | 14/29 (48.3%) | 6/7 (85.7%) | 0.104 | 0.369 |
| *optrA* | Antimicrobial resistance | Oxazolidinones | 2/32 (6.2%) | 0/7 (0.0%) | 1.000 | 1.000 | 2/29 (6.9%) | 0/7 (0.0%) | 1.000 | 1.000 |
| *lnu(E)* | Antimicrobial resistance | Lincosamides | 1/32 (3.1%) | 0/7 (0.0%) | 1.000 | 1.000 | 1/29 (3.4%) | 0/7 (0.0%) | 1.000 | 1.000 |
| *lnu(B)* | Antimicrobial resistance | Lincosamides | 1/32 (3.1%) | 0/7 (0.0%) | 1.000 | 1.000 | 1/29 (3.4%) | 0/7 (0.0%) | 1.000 | 1.000 |
| *lnu(C)* | Antimicrobial resistance | Lincosamides | 0/32 (0.0%) | 1/7 (14.3%) | 0.179 | 0.682 | 0/29 (0.0%) | 1/7 (14.3%) | 0.194 | 0.369 |
| *lsa(E)* | Antimicrobial resistance | Lincosamides | 1/32 (3.1%) | 0/7 (0.0%) | 1.000 | 1.000 | 1/29 (3.4%) | 0/7 (0.0%) | 1.000 | 1.000 |
| *cat* | Antimicrobial resistance | Chloramphenicol | 1/32 (3.1%) | 0/7 (0.0%) | 1.000 | 1.000 | 1/29 (3.4%) | 0/7 (0.0%) | 1.000 | 1.000 |
| *dfrG* | Antimicrobial resistance | Trimethoprim | 1/32 (3.1%) | 0/7 (0.0%) | 1.000 | 1.000 | 0/29 (0.0%) | 1/7 (14.3%) | 0.194 | 0.369 |

Notes: Virulence-associated genes were identified through VFDB screening and a literature-curated *S. suis*-specific reference dataset, whereas antimicrobial resistance genes were identified using ResFinder. For gene-level comparisons, Benjamini–Hochberg FDR correction was performed separately for the 70 virulence-associated genes and the 19 antimicrobial resistance genes. An FDR-adjusted *P* value <0.05 was considered statistically significant.

Abbreviations: ST, sequence type; FDR, false discovery rate; VFDB, Virulence Factor Database.
